# The role of NLRP3 inflammasome in stroke and central poststroke pain: Erratum

**DOI:** 10.1097/MD.0000000000013495

**Published:** 2018-11-21

In the article, “The role of NLRP3 inflammasome in stroke and central poststroke pain”,^[1]^ which appeared in Volume 97, Issue 33 of *Medicine*, the authors’ affiliation appeared incorrectly and should be “Department of Pain Management, Wuhan No. 1 Hospital, Wuhan, Hubei Province, China.”
